# Novel Insights into the Cardioprotective Effects of the Peptides of the Counter-Regulatory Renin–Angiotensin System

**DOI:** 10.3390/biomedicines12020255

**Published:** 2024-01-23

**Authors:** Janette Alejandra Gamiño-Gutiérrez, Ivana María Terán-Hernández, Jairo Castellar-Lopez, Wendy Villamizar-Villamizar, Estefanie Osorio-Llanes, Mariali Palacios-Cruz, Wendy Rosales, Aileen Y. Chang, Luis Antonio Díaz-Ariza, María Clara Ospino, Evelyn Mendoza-Torres

**Affiliations:** 1 School of Medicine, University of Guadalajara, Guadalajara 44360, Mexico; janettea-gaminog@unilibre.edu.co; 2 Grupo de Investigación Avanzada en Biomedicina, Faculty of Health Sciences, Universidad Libre Seccional Barranquilla, Barranquilla 081001, Colombia; ivanam-teranh@unilibre.edu.co (I.M.T.-H.); wendyp-villamizarv@unilibre.edu.co (W.V.-V.); luis-diazariza@unilibre.edu.co (L.A.D.-A.); mariac-ospinog@unilibre.edu.co (M.C.O.); 3 Grupo de Investigación Avanzada en Biomedicina, Faculty of Exact and Natural Sciences, Universidad Libre Seccional Barranquilla, Barranquilla 081001, Colombia; jairoa-castellarl@unilibre.edu.co (J.C.-L.); estefanie-osoriol@unilibre.edu.co (E.O.-L.); wendyd.rosalesr@unilibre.edu.co (W.R.); 4 Faculty of Medicine, Universidad Veracruzana, Veracruz 91090, Mexico; zs19009332@estudiantes.uv.mx; 5 School of Medicine and Health Sciences, The George Washington University, Washington, DC 20052, USA; chang@email.gwu.edu

**Keywords:** cardiovascular diseases, heart failure, renin–angiotensin system, angiotensin-(1-7), angiotensin-(1-9), alamandine

## Abstract

Currently, cardiovascular diseases are a major contributor to morbidity and mortality worldwide, having a significant negative impact on both the economy and public health. The renin–angiotensin system contributes to a high spectrum of cardiovascular disorders and is essential for maintaining normal cardiovascular homeostasis. Overactivation of the classical renin–angiotensin system is one of the most important pathophysiological mechanisms in the progression of cardiovascular diseases. The counter-regulatory renin–angiotensin system is an alternate pathway which favors the synthesis of different peptides, including Angiotensin-(1-7), Angiotensin-(1-9), and Alamandine. These peptides, via the angiotensin type 2 receptor (AT2R), MasR, and MrgD, initiate multiple downstream signaling pathways that culminate in the activation of various cardioprotective mechanisms, such as decreased cardiac fibrosis, decreased myocardial hypertrophy, vasodilation, decreased blood pressure, natriuresis, and nitric oxide synthesis. These cardioprotective effects position them as therapeutic alternatives for reducing the progression of cardiovascular diseases. This review aims to show the latest findings on the cardioprotective effects of the main peptides of the counter-regulatory renin–angiotensin system.

## 1. Introduction

Acute myocardial infarction (AMI), atherosclerosis, heart failure, valvular diseases, cardiac rhythm disorders, cerebrovascular disease, venous thromboembolism, pulmonary thromboembolism, arterial hypertension, and peripheral arterial disease are just a few of the pathologies that fall under the umbrella term of cardiovascular diseases (CVDs) [1,2]. It is essential to maximize our understanding of CVDs because they are all major global causes of morbidity and mortality and have a significant effect on public health and the economy [3]. According to estimates, 17.9 million people worldwide die from CVDs each year, making them the leading cause of death [4,5,6].

According to the American Heart Association, there are around 2380 deaths caused by CVD every day and one person dies from one of these conditions in the US every 36 s [6]. In Mexico, the statistics presented by the National Institute of Statistics and Geography show that from January to August 2020, 141,873 people died from CVD, a number that even exceeded deaths from COVID-19 [7]. Over time, and with the exponential growth of scientific advances, the pathophysiological mechanisms involved in the progression of CVD have been determined [8]. In Colombia, the vital statistics registered by the National Administrative Department of Statistics show that during 2021, 55,717 people died from CVD, and of these deaths, 47,621 were due to AMI, 4589 to primary hypertension, and 3507 to hypertensive heart disease with heart failure [9]. 

Under physiological conditions, the renin-angiotensin system (RAS) [10] participates in the regulation of cardiac output, vasoconstriction, hydroelectrolytic balance, cell growth, and the integrity of the vascular wall. However, under pathological conditions, the overactivation of this system promotes the development of acute or chronic CVD through mechanisms of proliferation, remodeling, and inflammation [11,12]. The RAS is a pathway mediated by multiple enzymatic reactions that produce a wide variety of peptides. This system starts with the renin produced in the juxtaglomerular cells of the kidney, which then cleaves angiotensinogen from the liver [13] to form angiotensin I (Ang I). Later, angiotensin converting enzyme (ACE) cleaves Ang I to convert it into angiotensin II (Ang II), which can bind to the angiotensin type II receptor (AT1R) to favor increases in total peripheral resistance (TPR), the production of aldosterone, and peripheral vasoconstriction which increases arterial pressure [14]. It was previously believed that the product of the system, Ang II, was the main and only functional peptide and primary ligand of the AT1R and that its binding primarily regulates blood pressure control [15].

Currently, it has been described that the RAS is more complex and that the products generated in the pathway are potentially functional since its target receptors determine different effects that are important objects of study for a better understanding of the system, its role as a pathophysiological determinant of CVD, and its importance as a future target and therapeutic resource [10].

Angiotensin-(1-7) (Ang-[1-7]), angiotensin-(1-9) (Ang-[1-9]), and alamandine are three peptides of the counter-regulatory RAS that have shown cardioprotective effects [12,16,17]. Ang-(1-7), Ang-(1-9), and alamandine bind to the Mas receptor (MasR), AT2R, and MrgD, respectively, promoting ligand/receptor binding and antagonizing the effects of Ang II, including vasodilator, antihypertensive, and antifibrotic effects [18,19,20]. The objective of this review is to present the latest evidence supporting the cardioprotective effects of Ang-(1-9), Ang-(1-7), and alamandine.

## 2. Cardiovascular Diseases

**Figure 1:**
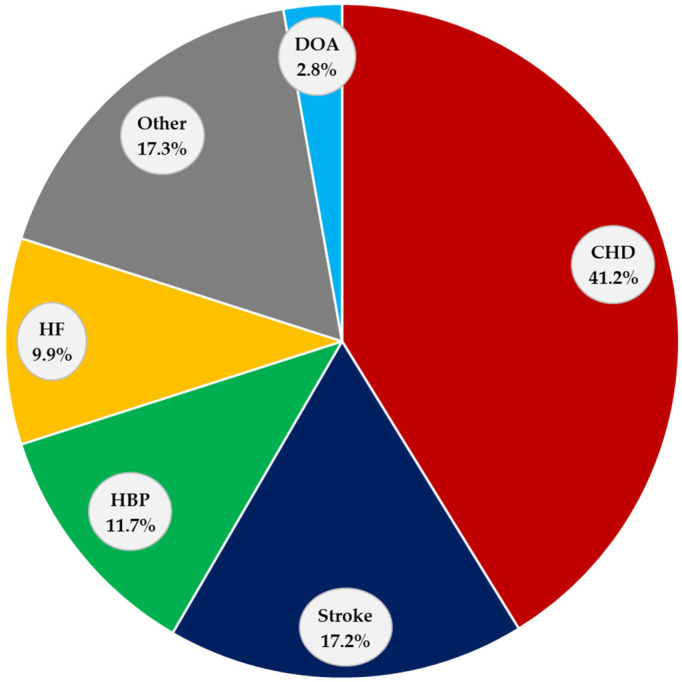
Causes of CVD death. CVD: cardiovascular disease, CHD: coronary heart disease, Other: other minor CVDs combined, HBP: high blood pressure, HF: heart failure, and DOA: diseases of the arteries.

CVDs are the leading cause of death, disability, and medical spending globally [21,22]. The American Heart Association reports that coronary heart disease (CHD) is by far the main cause of death (41.3%), followed by stroke (17.2%), high blood pressure (HBP) (11.7%), heart failure (HF) (9.9%), diseases of the arteries (2.8%), and other minor CVD causes combined (17.3%) (Figure 1) [23]. 

### 2.1. Coronary Heart Disease (CHD)

Coronary heart disease occurs when there is an imbalance between the supply and demand of cardiac tissue. The demand corresponds directly to the amount of blood supply required by the myocardium, while the supply refers to the amount of blood that perfuses this tissue [24]. Coronary blood flow can be reduced by the presence of atheromatous plaques or by different degrees of vasoconstriction present in the coronary arteries [25]. Coronary heart disease thus influences the development of different heart diseases.

The main cause of CHD is AMI, which is defined according to the fourth universal definition as the presence of cardiomyocyte necrosis in a clinical context consistent with acute myocardial ischemia, detected by a rise or fall of hs-cTn (high-sensitivity cardiac troponin) values with at least one value above the 99th percentile [26,27], with symptoms of myocardial ischemia (chest pain radiating from the left arm to the neck, dyspnea, sweating, nausea, vomiting, abnormal heartbeats, anxiety, fatigue, and weakness, among others [28,29]), changes in the electrocardiogram (ECG), image of evident myocardial damage, or the finding of coronary thrombus at autopsy.

During AMI, the hypoxic environment generated by the absence of irrigation causes ionic and metabolic alterations in the tissue, causing the subsequent necrosis of cardiomyocytes [27,30]. After ischemia, and secondary to the release of mediators, the inflammatory phase is established in the injury area [31]. Once completed, the proliferation phase begins which is characterized by an increase in the population of myofibroblasts with the subsequent production of fibers and contractile proteins that contribute to the process of remodeling and repair of the injured myocardium [32,33].

### 2.2. Stroke

Stroke is caused by a decrease in cerebral flow that causes neuronal injury. Each year, 795,000 individuals in the United States experience a stroke, of which 87% (690,000) are ischemic and 185,000 are recurrent [34]. There are three main causes of stroke. The first one is the presence of atheromatous plaques in the cerebral vasculature. The second cause is cerebral infarction of cardiogenic origin, and the final cause is the presence of lacunar infarcts due to injury of small vessels [35]. There are different pathophysiological mechanisms including a decrease in flow conditions, a lower supply of glucose to the tissue, alteration in the synthesis of ATP, the development of edema, inflammation, and neuronal death, thus contributing to the development of neurological deficits [36].

### 2.3. High Blood Pressure (HBP)

There are multiple pathophysiological mechanisms that cause the development of arterial hypertension. These include mechanisms involving the RAS, natriuretic peptides, factors of the vascular endothelium, and the central nervous system [37]. High blood pressure is one of the main risk factors that contributes to the development of cardiovascular disease [38]. The primary cardiovascular disease associated with high blood pressure is acute myocardial infarction [39]. There is a strong association between hypertension and the development of heart failure, secondary to cardiac remodeling, diastolic dysfunction, and alterations in the ejection fraction [40]. 

### 2.4. Heart Failure (HF)

Heart failure (HF) is often caused by a structural or functional cardiac disorder, such as AMI, which is the leading cause of HF in the world, that leads to a reduction in heart rate and that causes an alteration in cardiac output. Patients with HF report orthopnea, paroxysmal nocturnal dyspnea, and edema of the lower extremities. On physical examination, it is common to find elevated jugular venous pressure and pulmonary congestion [41,42,43,44].

Heart failure in patients hospitalized for AMI has a frequency of presentation ranging from 14% to 36% [45]. In fact, it is mandatory to perform the Killip–Kimbal classification according to the presence of signs of HF in patients with AMI [46]. As previously mentioned, after an AMI there is a remodeling process in the cardiac tissue [47,48]. HF after AMI is a consequence of cardiomyocyte death, the formation of scars by fibroblasts that repair the necrotic area, and adaptive effects that are activated to maintain adequate hemodynamic function [49,50,51].

The most common cause of HF in the world is related to an AMI. After the initial insult, an inflammatory phase occurs; then, a repair and proliferation phase with subsequent myocardial remodeling, which may result in mechanical, electrical, or hemodynamic alterations, which leads to deterioration in the patient’s quality of life, increased prescriptions of medications, and more admissions to hospitalization. This may explain the great economic and social impact that AMI has on the population.

### 2.5. Other CVDs

There are other minor CVDs that are worth mentioning in this review. These can appear as a direct complication of major cardiovascular disease [23].

Heart rhythm disorders are a common condition. It is known that the main risk factor for developing atrial fibrillation is the presence of high blood pressure. There are other situations such as AMI that are directly associated with the appearance of rhythm disorders such as ventricular arrhythmias [52].

Valvular diseases are similarly correlated with high blood pressure. According to the American Heart Association, patients with high blood pressure have an odds ratio (OR) of 1.71 (95% CI) of developing aortic stenosis [23,53]. Similarly, high blood pressure correlates with the development of peripheral arterial disease (OR 1.47 CI 95%) and is considered one of the main preventable risk factors [23]. 

## 3. Renin–Angiotensin System (RAS)

The RAS has been studied for a long time, due to its fundamental role in regulating the functioning of the cardiovascular system [54]. The role of the classical RAS in the regulation of renal and cardiovascular physiology has been widely studied, which has also led to the identification of its implications in the pathological context. However, recently new components have been identified that constitute a noncanonical and counter-regulatory RAS that antagonizes the deleterious effects of the classical RAS. Further research is needed to determine therapeutic targets in the counter-regulatory RAS.

### 3.1. Classical RAS

The classical RAS begins with the production of pre-prorenin in the juxtaglomerular cells of the kidney, which is cleaved to form prorenin prior to becoming active renin. Renin is released into the systemic circulation under specific stimuli, such as low systemic blood pressure, situations of hypovolemia of any etiology, and decreased sodium or sympathetic stimulation [54].

Renin is then responsible for metabolizing angiotensinogen, a peptide that is released from the liver into the circulation, to thereby produce the decapeptide Ang I. The next step in the pathway is mediated by ACE, which is synthesized in the endothelium and cleaves Ang I to remove two amino acids to generate Ang II [55,56]. Ang II binds to the AT1R receptor, causing increased sympathetic tone, decreased parasympathetic tone, decreased baroreflex sensitivity, increased blood pressure, vasoconstriction, decreased nitric oxide (NO) synthesis, aldosterone, and antidiuretic hormone release (ADH); Ang II also promotes myocardial hypertrophy, increases cardiac fibrosis, has a proinflammatory effect, increases the production of reactive oxygen species (ROS), and decreases natriuresis. In addition to Ang I and II, other peptides are generated in the RAS that were previously believed to have no function and were considered metabolic residues. The knowledge of these peptides helped the discovery of an alternative pathway dependent on the classical pathway of the RAS, which is known as the counter-regulatory RAS [57].

### 3.2. Counter-Regulatory RAS

**Figure 2:**
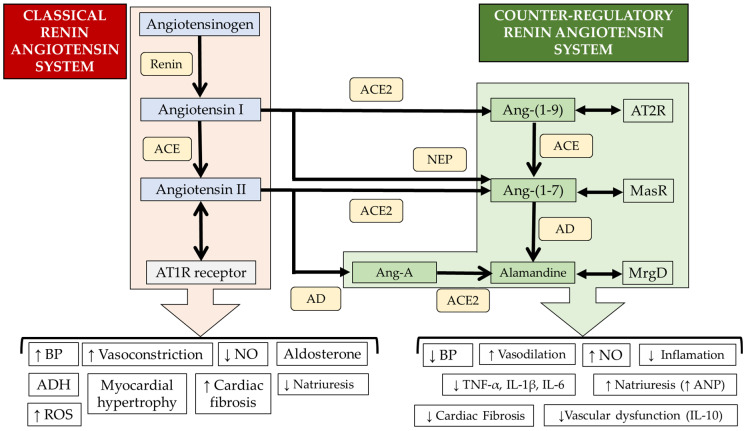
Classic and counter-regulatory renin–angiotensin systems. ACE: angiotensin-converting enzyme, AT1R: angiotensin II type 1 receptor, BP: blood pressure, ADH: antidiuretic hormone, ROS: reactive oxygen species, NO: nitric oxide, NEP: neprilysin, AD: aspartate decarboxylase, MasR: Mas receptor, MrgD: Mas-related G protein-coupled receptor D, AT2R: angiotensin II type 2 receptor, TNF-α: tumor necrosis factor-alpha, IL-1β: interleukin-1 beta, IL-6: interleukin-6, ANP: atrial natriuretic peptide, IL-10: interleukin-10, and Ang-A: angiotensin A. ↑: increase; ↓: decrease.

The counter-regulatory RAS includes Ang-(1-9), Ang-(1-7), alamandine, Ang A, and the enzymes that are responsible for their synthesis. Ang-(1-7) is synthesized by cleavage of Ang I and Ang II by neprilysin (NEP) and ACE-2, respectively. Ang-(1-9) is produced by cleavage of Ang I by ACE2 and can be cleaved by ACE to produce Ang-(1-7) which can be metabolized to alamandine via decarboxylation of the N-terminal aspartic acid catalyzed by aspartate decarboxylase (AD). On the other hand, Ang II is processed by aspartate decarboxylase (AD) to produce Ang A, which can be cleaved to alamandine by ACE [57] (Figure 2). These peptides of the counter-regulatory RAS have beneficial effects on the cardiovascular system and are considered as potential therapeutic alternatives because they antagonize the pathological effects of overactivation of the ACE/Ang II/AT1R axis which is part of the classical RAS.

#### 3.2.1. Ang-(1-7)

Ang-(1-7) is a heptapeptide of the counter-regulatory RAS with various local and systemic effects that counteract the effects of Ang II [58]. Robson et al. demonstrated the specificity of Ang-(1-7) for MasR. In their experiments, I^125^ was linked to Ang-(1-7) and exposed to mouse cells with and without MasR deficiency. Specific binding of I^125^-Ang(1-7) was significantly higher in kidneys of wild type compared to MasR-deficient mice [59]. Ang-(1-7) is produced from Ang II by ACE2 [60,61]. It has been demonstrated that Ang I can also be transformed into Ang-(1-9) by ACE2 and then cleaved by ACE or NEP to create Ang-(1-7), but with less effectiveness [62]. Since 2003, it has been proven that Ang-(1-7) binds specifically to MasR [61]. 

Studies on animal models have shown that Ang-(1-7) exerts vasodilator, anti-inflammatory, and antifibrotic effects by activating signaling pathways associated with the synthesis of NO [60,62], inhibition of MAP kinase signaling (ERK1/2, p38, and JNK), ROS production by NADPH oxidases and transforming growth factor beta–SMAD protein (TGF-β-SMAD), and signaling and modulation of the cyclic adenosine monophosphate (cAMP) signaling response [60]. 

Ang-(1-7) plays a cardioprotective role, since it influences the development of antifibrotic, antihypertrophic, and antiarrhythmogenic effects, preventing the proliferation of cardiomyocytes and promoting the synthesis of NO. In addition, Ang-(1-7) is involved in protection against cardiac hypertrophy and Ang II-induced fibrosis by reducing oxidative stress and collagen deposition and increasing atrial natriuretic peptide (ANP) secretion [62].

On the other hand, according to a study carried out by Freitas RA et al., synergistic action between Ang-(1-7) and interleukin 10 (IL-10) was demonstrated, which converge in a mechanism protective against vascular dysfunction, because they exert on the vascular contractility response [63]. This research was conducted in order to test the idea that IL-10 has an immunomodulatory effect on the vasculature and that Ang(1-7) also has modulatory effects that are advantageous in many tissues. However, given that most of the research focuses on demonstrating the preventive cardiometabolic benefits in young animals, it is interesting to highlight a study conducted in 2020 that sought to evaluate the actions of Ang-(1-7) affecting the circulatory system in aged animals [64]. Infusion of Ang-(1-7) improved the cardiometabolic function, specifically systolic blood pressure, mean blood pressure, and insulin sensitivity [64]. These lowering effects were associated with reduced measures of the cardiac sympathetic tone. Ang-(1-7) has been classified as a new promising target for the treatment of heart failure in conjunction with Ang-(1-9) due to its function in cardiac remodeling and counter regulation of the detrimental effects of Ang II [65].

Ang-(1-7) has cardiac effects due to its antihypertrophic, antifibrotic, and antiarrhythmogenic properties and peripheral effects by improvement in vascular contractility mediated by immunomodulation, contributing to the reduction in ventricular remodeling and positively impacting on cardiac function. All these effects make Ang-(1-7) a potential therapeutic alternative that could improve the quality of life of patients after AMI.

#### 3.2.2. Ang-(1-9)

RAS dysfunction has a significant impact on the development of cardiovascular disease [66]. The octapeptide Ang II, which regulates blood pressure and aldosterone secretion, is one of the components of the traditional RAS [67]. Most of the physiological and pathological effects of Ang II in the cardiovascular system are mediated by the AT1R [68]. Ang-(1-9) is a peptide of the counter-regulatory RAS that counteracts the physiological and pathological actions of Ang II [69]. Flores et al. (2011), with radioligand binding assays, demonstrated a specificity of Ang-(1-9) for the AT2R receptor, reporting that Ang-(1-9) could bind the AT2R (p K i = 6.28 ± 0.1), concluding that there is an important direct biological role for Ang-(1-9) acting through AT2R [70].

Recent data have shown that Ang-(1-9) protects the heart and blood vessels from adverse cardiovascular remodeling in experimental models of hypertension and/or ischemia/reperfusion (I/R) events and reduces cardiac fibrosis in spontaneously hypertensive rats prone to stroke. These effects are mediated by the AT2R [69]. This peptide can increase the plasma fibrinogen level, increasing the generation of fibrin, similarly to Ang II [71]. 

Ang-(1-9) has a protective effect on the heart and blood vessels against I/R injury, which could have promising results as a therapeutic target for the induction of reverse ventricular remodeling in patients with heart failure and coronary artery disease.

#### 3.2.3. Alamandine

Alamandine is a recently identified heptapeptide that is a product of the decarboxylation of Ang-(1-7) [72]. This peptide is structurally similar to Ang-(1-7) and differs in a single N-terminal alanine residue [73] instead of an aspartate residue [74,75]. Alamandine shows an affinity for MrgD, a member of the Mas-related G protein-coupled receptor (MRGPR) family. The MrgD receptor has 42% sequence similarity and 20% identity within the seven-transmembrane domain of the AT1R receptor [76]. Lautner et al. used almandine labeled with fluorescein to test its specific binding to MrgD in Chinese hamster ovary (CHO) cells transfected and not transfected with MrgD. Specific union with MrgD and almandine was determined [77]. 

The alamandine/MrgD complex initiates a downstream signaling pathway that significantly reduces the levels of TNF-α, IL-1β, IL-6, and NO [78]. Alamandine has several physiological effects that are similar to those of Ang-(1-7), including vasodilation, antifibrosis, and blood pressure lowering. Its actions are independent of the recognized vasodilator receptors of the RAS and MasR receptors, according to current research [73,74].

## 4. Cardioprotective Effects of the Peptides of the Counter-Regulatory RAS

### 4.1. Cardioprotective Effects of Ang-(1-7)

**Figure 3:**
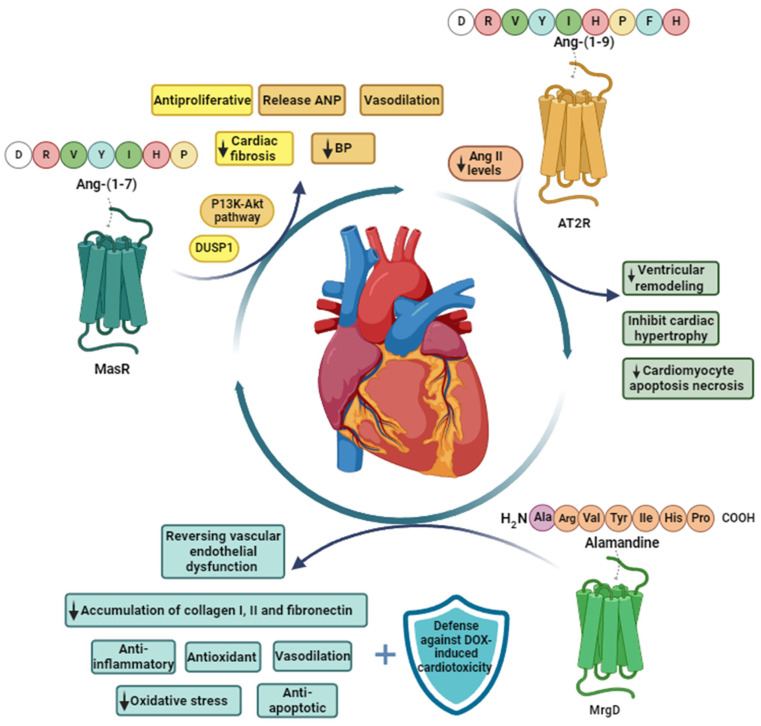
Cardioprotective effects of Ang-(1-9), Ang-(1-7), and alamandine. AT2R: angiotensin II type 2 receptor, Ang II: angiotensin II, BP: blood pressure, MasR: Mas receptor, and ANP: atrial natriuretic peptide. ↑: increase; ↓: decrease.

As previously stated, Ang-(1-7) acts via MasR [79], a G protein-coupled receptor (Figure 2). After binding of Ang-(1-7) to MasR, multiple signaling pathways are activated that generate cardioprotective effects and attenuate the effects induced by Ang II [79,80]. Figure 3 shows the signaling and cardiac effects of Ang-(1-7).

Ang-(1-7) promotes vasodilation via the activation of the MasR through the NO–soluble guanylyl cyclase pathway, opposing the effects produced by Ang II [81]. Carver et al. demonstrated that Ang-(1-7) increases DUSP1 (dual-specificity phosphatase 1), to reduce MAP kinase/Smad/CTGF signaling and consequently decrease fibrosis in resistance arterioles [82]. Wang LP et al. (2016) showed for the first time that the ACE2–Ang-(1-7)–MasR axis significantly prevented cardiac fibrosis in murine models by inhibiting the effect on KCa3.1 channels through the ERK1/2 pathway [83]. In hypertensive two-kidney rats trained with a single clip, administration of Ang-(1-7) decreased fibrosis and was accompanied by upregulation of MasR and phosphorylation of eNOS [2,84]. 

The regulation of several extracellular matrix proteins may also play a role in the antiproliferative and antifibrotic action of MasR activation in the heart [85]. Ang-(1-7) decreased TGF-β1 mRNA levels in cultured cardiac fibroblasts [86], reduced plasma TGF-β1 levels in a rat model of myocardial infarction [87], and increased vascular remodeling [88]. Oral treatment with the nonpeptide Ang-(1-7) analog, AV0991, reduces cardiac remodeling in hypertensive rats [89]. In cardiac fibroblasts, Ang-(1-7) reduced DNA, protein, and collagen synthesis after stimulation with serum and endothelin-1 [90]. 

Recently, it has been shown that a formulation including Ang-(1-7) in hydroxypropyl β-cyclodextrin produced an improvement in diastolic and systolic functions and reduced the expression of fibrosis scar markers (TGF-β and type I collagen) in a rat model of myocardial infarction induced by left coronary artery occlusion [91]. In endothelial cells, Ang-(1-7) was discovered to be an antisenescence peptide that functions independently of the RAS by concurrently activating klotho and Nrf2/HO-1. Therefore, avoiding endothelial cell senescence and its associated vascular problems may be possible with the use of Ang-(1-7) mimetic drugs [92]. Ang-(1-7) enhanced collagen deposition in vivo. Ang-(1-7) decreased the expressions of collagen type I and α-SMA and increased the expressions of ACE2 and MasR in silicotic rat lung tissue and fibroblasts stimulated by Ang II. Ang-(1-7) exerted a protective effect on Ang II-induced myofibroblast differentiation and silicotic fibrosis by regulating the ACE2–Ang-(1-7)–MasR axis [93]. Chronic infusion of Ang-(1-7) into the brain modulates inflammatory mediators, RAS components, and iNOS in the hypothalamus, suggesting a possible additional antihypertensive mechanism of Ang-(1-7) in the central nervous system [94]. Stoyell-Conti et al. compared HR in a saline-infused control group versus a group administered Ang-(1-7) as well as iodine Ang-(1-7). Ang-(1-7) and iodoAng-(1-7) prevented the increase in HR in the saline group during the protocol. The iodoAng-(1-7) group had a lower heart rate than the saline group [95]. Ang-(1-7) could be a recurrent additive in heart surgery and cardioprotection strategies.

The cardioprotective effects of Ang-(1-7) are explained by multiple pathways, one of which is the PI3K–Akt signaling pathway that can induce ANP secretion and vascular regulation mediated by IGF-1 and VEGF; it also decreases microvascular fibrosis through the DUSP1 pathway and its effects on TGF-β1 by reducing ventricular remodeling [91,92,93,94,95].

### 4.2. Cardioprotective Effects of Ang-(1-9)

Ang-(1-9) exerts cardioprotective effects via AT2R, a G protein-coupled receptor [79] (Figure 2). Ang-(1-9) is a relatively more stable intermediate than Ang-(1-7) in terms of ACE2 regulation and is also thought to reduce Ang II levels because it competes with Ang I at the active site of ACE, increases Ang-(1-7), and stimulates bradykinin release from endothelial cells [79]. Ang-(1-9) can inhibit cardiac hypertrophy after myocardial infarction, attenuates cardiac fibrosis in stroke-prone spontaneously hypertensive rats and prevents cardiomyocyte hypertrophy by regulating mitochondrial dynamics [17,96,97]. Furthermore, this peptide also prevents vascular remodeling by a Foxo1-dependent mechanism [98] and decreases cardiomyocyte death during simulated I/R [99]. These findings show the potential of Ang-(1-9) to improve recovery of the cardiac function after AMI or cardiac surgeries in which the use of cardioplegia is necessary [100,101]. Both Ang-(1-9) and Ang-(1-7) may prevent cardiomyocyte hypertrophy and pathological ventricular remodeling [101,102,103]. 

When Ang I was injected into the pulmonary and renal arteries of dogs, it was discovered that leucine quickly began to manifest. This discovery provided the first proof for this peptide. The authors suggested that a carboxypeptidase was involved in the catalysis of Ang I to form a compound known at the time as des-Leu10 Ang I [104] now known as Ang-(1-9). Initially, it was thought that this peptide lacked biological activity and only indirect action was attributed to it due to its ability to compete with Ang I for the active sites of ACE, generating high levels of Ang-(1-7) and decreasing the Ang II [105]. However, in recent years, evidence of a direct effect of Ang-(1-9) on the cardiovascular system has increased [106]. 

Sotomayor Flores-C et al. confirmed that changes in mitochondrial dynamics and Ca^2+^ handling are related to the development and progression of pathological cardiac hypertrophy. They also showed for the first time that Ang-(1-9) controls this mitochondrial process in a model of neonatal rat cardiomyocytes, upregulating miR-129-3p and inhibiting protein kinase A inhibitor (PKIA). Ang-(1-9) also inhibits mitochondrial fission, a crucial process underlying its pro-hypertrophic actions, as previously mentioned [97]. This has been very beneficial for research because, at the cellular level, cardiac hypertrophy is associated with metabolic changes that are specifically linked to the alteration of mitochondrial function and morphology, because this mitochondrial function supports the modulation of intracellular Ca^2+^ signaling, which in turn affects changes in gene and protein expression, among other processes, and helps to satisfy the high demands of energy. The importance of this organelle in preserving good cardiac function is highlighted by the fact that approximately 70% of ATP synthesis takes place in the mitochondrial matrix, highlighting its prominent involvement in the heart [97]. Norambuena-Soto et al. also conducted a study to evaluate the anti-vascular-remodeling effects of Ang-(1-9), giving special focus to the control of the vascular smooth muscle cell phenotype. Through this research, they showed that Ang-(1-9) decreased blood pressure and aortic thickness in spontaneously hypertensive rats and that the same thickness reduction was associated with decreased smooth muscle cell proliferation. In addition, Ang-(1-9) treatment inhibited migration and decreased the effects induced by platelet-derived growth factor-BB [98]. Mendoza Torres et al. showed that Ang-(1-9) limited reperfusion-induced cell death via an AT2R and Akt dependent mechanism [99]. Figure 3 shows the signaling and cardiac effects of Ang-(1-9). 

Protein kinase B (Akt) is a crucial component of the cell cycle process. It has been linked to cancer, cell growth, inactivity, and aging [107]. Activation of PI3K further phosphorylates and activates Akt, which localizes to the cell membrane and once activated serves a variety of functions, including activation of cAMP-response element-binding protein, inhibition of p27, localization of protein O in the cytoplasm, and the activation of phosphatidylinositol 3-phosphate, among others [107]. The PI3K/Akt signal transduction pathway, which involves the activation of PI3K, results in the binding of Akt to the cell membrane with the aid of phosphoinositide-dependent kinase. However, Akt is activated because of threonine and serine phosphorylation, which also mediates other enzymatic biological effects such as those related to cell growth, apoptosis suppression, cell migration, vesicle transport, and transformation cell malignancy [107]. Recent research on the signaling pathways that are activated by the counter-regulatory RAS has highlighted it as a potential therapeutic target for the management of CVD [17]. Ang-(1-9) has been linked to the PI3K/Akt signaling system; evidence suggests that it can stimulate ANP secretion activating the endothelial PI3K–Akt–NO synthase pathway [17]. The interaction of Ang-(1-9) with AT2R may activate Akt signaling pathway leading to natriuresis and NO production, which promotes vasodilatory effects and lowers blood pressure [17]. Mendoza Torres et al. showed that Ang-(1-9) decreases apoptosis and necrosis in neonatal rat cardiomyocytes subjected to simulated I/R and improves left ventricular function and decreases infarct size in isolated rat hearts through an AT2R and Akt-dependent mechanism [99]. The PI3K–Akt signaling pathway associated with insulin-like growth factor (IGF-1) is considered one of the key molecular mechanisms responsible for exercise-induced cardiac growth and protection, considering it to be the primary signaling pathway in the physiological onset of cardiac hypertrophy [108]. Activation of this signaling cascade has also been shown to protect the heart in mouse models of cardiac injury and cardiovascular disease, whereas reduced IGF1–PI3K–Akt signaling is detrimental to cardiac function and accelerates disease progression [17]. Another important function that has been associated with Akt is in the process of angiogenesis through the regulation of the NO signaling pathway. The PI3K/Akt pathway releases a group of angiogenic factors including vascular endothelial growth factor (VEGF) [81]. Receptor 2 for VEGF has a central role in angiogenesis which, in turn, is required for endothelial cell migration and enables the formation of capillary-like structures through a PI3K/Akt-dependent manner. NO is released from the endothelium and represents the main mediator of smooth muscle tone causing vasodilation through endothelial-type nitric oxide synthase activity [81]. For all the above, the important role that PI3K/Akt signaling pathway plays as a regulatory mechanism in physiological processes and the disadvantages that its deregulation entails are made evident. Furthermore, the evidence that demonstrates the influence of Ang-(1-9) on the regulation of Akt promotes the development of studies to explore other effects that this peptide may exert through the PI3K/Akt signaling pathway.

The mechanisms by which Ang-(1-9) exerts its beneficial effects on the heart are explained by the regulation of intracellular calcium and the inhibition of mitochondrial fission, crucial processes in cardiomyocyte hypertrophy.

### 4.3. Cardioprotective Effects of Alamandine

Alamandine is an endogenous ligand of MrgD [72,79]. This has been supported by a study in Wistar rats treated with isoproterenol, where oral administration of alamandine contained in 2-hydroxypropyl-cyclodextrin reduced the accumulation of collagen I and III, as well as fibronectin, in cardiac tissue of rats treated with isoproterenol [77,109]. In another study conducted in male C57BL/6J mice, which were divided into three groups, namely, (1) Simulated surgery, (2) Operated, and (3) Operated + treated with alamandine-HPβCD (2-hydroxypropyl-β-cyclodextrin, 30 μg/kg/day, via probe), administration of alamandine for 14 days decreased arterial remodeling, resulting in a reduction in the thickness of the middle layer of the ascending aorta, along with an attenuation of fibrosis at this same level and reduction in the vascular expression of proinflammatory genes (CCL2, TNF-α, IL-1β) [110]. Lautner et al. demonstrated that alamandine prevented the development of cardiomyocyte hypertrophy and the evolution to cardiac fibrosis and decreased molecules that are activated during cardiac remodeling [77]. Treatment with alamandine, 20 min before ischemia, reduced the infarct size, apoptotic proteins, and LDH levels in isolated rat hearts subjected to global ischemia for 20 min and 50 min of reperfusion [77]. 

On the other hand, alamandine exerts antioxidant, anti-inflammatory, and antiapoptotic effects, and is a cardioprotective alternative against doxorubicin (DOX)-induced cardiotoxicity [110]. Hekmat et al. discovered that the administration of alamandine restored the alterations induced by DOX in the cardiac muscle as well as at the level of vascular congestion, relieving effects such as cardiac contractility, decreased systolic and diastolic blood pressure, and increased left ventricular end-diastolic pressure. Alamandine also decreased oxidative stress and inflammatory cytokines [110,111]. Furthermore, alamandine counteracted the hypertrophic effects generated by Ang II in isolated ventricular cardiomyocytes from C57BL/6 mice through activation of the AMPK/NO signaling pathway via MrgD [75]. In a mouse model of heart failure induced by myocardial infarction, alamandine was administered via intraperitoneal injection for two weeks, resulting in a reduction in fibrosis, oxidative stress, apoptosis biomarkers, and cardiac dysfunction [112].

The N-terminal Asp/Ala amino acid is the only difference between alamandine and Ang-(1-7). This could account for the striking similarity of most of the biological impacts examined. Alamandine can cause endothelium-dependent vasodilation in the aortic rings of mice and rats as has been demonstrated with Ang-(1-7) [113]. Additionally, alamandine microinjections generated cardiovascular effects comparable to those of Ang-(1-7): elevated blood pressure in the rostral ventrolateral medulla oblongata and lowered blood pressure in the caudal ventrolateral medulla oblongata [77]. Although alamandine and Ang-(1-7) have comparable actions, their respective receptors are different [114]. In addition to the cardioprotective effects, alamandine exerted protective effects at the renal level against I/R injury. In fact, alamandine improved renal function, and attenuated inflammation and apoptosis, and decreased serum levels of creatinine, blood cystatin C, and blood urea nitrogen, as well as oxidative stress [114]. Figure 3 shows the impact of alamandine on cell signaling in cardiovascular signaling.

Alamandine is a molecule similar to Ang-(1-7) that appears to prevent the development of myocardial hypertrophy and fibrosis, in addition to having anti-inflammatory and antioxidant effects. It has become an important focus in the understanding of the cardioprotective and reno-protective mechanisms of the alternative pathways of the RAS. Preclinical studies with almandine for damage reduction and myocardial fibrosis have shown promising results, which opens the door for clinical studies to corroborate these effects in humans. 

Table 1 summarizes the main cardioprotective effects of Ang-(1-7), Ang-(1-9), and alamandine. 

## 5. Clinical Trials with the Peptides of the Counter-Regulatory RAS 

In the race to make the leap from the bench to the bedside, Ang-(1-7) has certain advantage over Ang-(1-9), and alamandine. This may be due to the fact that the functional role of Ang-(1-7) in the antagonism of the effects of Ang II was known before the discovery of ACE2 [115] and initially Ang-(1-9) was thought to be biologically inactive, operating indirectly by competing with Ang I for the ACE and leading to reduction in Ang II levels while increasing those of Ang-(1-7) [105,116]. It took several years for evidence to appear showing that Ang-(1-9) exerts direct cardiovascular effects through AT2R [96,102,117]. Furthermore, the low plasma levels for Ang-(1-9), ranging from 5 to 50 fmol/mL [118,119], raise suspicions of its low half-life which is up to now still unknown. However, clinical trials listed on www.clinicaltrials.gov explore the safety of other alternatives for AT2R agonists, such as LP2 and C21 and recombinant ACE2 (rACE2), but no clinical trials have been found that study the direct effects of Ang-(1-9) or a more stable pharmacological alternative of this peptide. The safety and tolerability of rACE2, GSK2586881 from GlaxoSmithKline, and APN01 from Apeiron Biologics, have been evaluated in two double-blind, placebo-controlled dose-escalation phase 1 clinical trials. rACE2 was shown to be safe and well tolerated [120,121].

On the other hand, there is a phase 1, open-label, single-center clinical trial that evaluated the effects of C21 on forearm blood flow in healthy male subjects by use of strain-gauge venous occlusion plethysmography but the results have not yet been published [122]. To date, there are no clinical trials related to alamandine due to its recent discovery and the lack of greater preclinical evidence to continue the evaluation of its effects in patients [111].

Due to the cardioprotective effects demonstrated in preclinical models, Ang-(1-7) has become a therapeutic target for reducing cardiovascular risk, especially in scenarios of heart failure and ischemic heart disease. This has motivated the conduct of clinical trials aimed at demonstrating its beneficial effects on the cardiovascular system. While most of these trials are still ongoing, it is expected that the results will shed light on a new therapeutic arsenal that can help decrease the overall burden of cardiovascular disease. 

A total of 24 interventional clinical trials with Ang-(1-7) were found in the literature, of which 8 correspond to the evaluation of its cardiovascular benefits; Four clinical trials are in the recruitment phase (phase 1 and 2), two in active status, one in completed status and one was in withdrawn status due to discontinuation of the drug for intervention.

Considering that aging is one of the risk factors for the development of cardiovascular diseases, there is a study (NCT05301192, recruitment status phase 1) that seeks to demonstrate the effect of Ang-(1-7) on sympathetic nervous system activity to restore β2 vascular adrenergic receptor signaling, which may help reduce cardiovascular risk during aging. Participants in this clinical trial will receive Ang-(1-7) intravenously for 110 min, administered in escalating doses of 2 ng/kg/min, 4 ng/kg/min, and 8 ng/kg/min (each of these doses will be infused for 10 min). After the dose escalation, Ang-(1-7) will be administered at 8 ng/kg/min for an additional 80 min. Blood and endothelial cell samples are taken before and after each infusion to measure endothelial function [123]. 

Another clinical trial (NCT03604289, recruitment status early phase 1) aims to assess the effect of Ang-(1-7) in patients with obesity and hypertension, intending to demonstrate whether there is an improvement in cardiovascular health in this group of patients [124].

Another clinical trial (NCT03240068, active status) aims to determine the cardiovascular effects of Ang-(1-7) in patients with peripheral artery disease (men or women between 21 and 80 years old) through acute intravenous infusions (five escalating doses of 10 min each: 1, 2, 4, 8, and 12 ng/kg/min). They will evaluate whether Ang-(1-7) leads to improvements in lower limb blood flow and reduces systemic inflammatory action in participants [125].

An open multicenter clinical trial is currently being conducted, in phase 2 recruitment (NCT06013839), that evaluates its usefulness and effectiveness in patients with cardiomyopathy related to Duchenne muscular dystrophy. In this study, the change in ejection fraction will be evaluated at 6 and 12 months in patients who received an Ang-(1-7) regimen at a dose of 0.5 mg/kg/day for 6 months [126].

In the context of strokes, a double-blind, randomized, placebo-controlled, hybrid decentralized study (NCT06135103; recruitment status phase 2) aims to evaluate the safety and efficacy of daily subcutaneous injection of Ang-(1-7) in post-ischemic stroke patients to improve their motor and sensory functions as measured with the Fugl-Meyer Assessment of Upper Extremity (FMA-UE). Subjects will receive either Ang-(1-7) 0.5 mg/kg or a placebo for 12 weeks started 6 to 24 months post ischemic stroke, and they will have a 12-week follow up visit after treatment has ended [127]. 

Likewise, a study carried out in 2022 evaluated the cardioprotective effects of counterregulatory peptides in diabetic patients through endomyocardial biopsies. The study reported that patients with poor metabolic control presented non-enzymatic glycation of four lysine residues of the ACE2 receptor (GlycACE2). An interesting finding was an inverse relationship between the presence of GlycACE2 and Ang-(1-7), Ang-(1-9), and MasR levels (Ang 1-7: R = −0.844, *p* < 0.001; Ang 1-9: R = −0.762, *p* < 0.001; MasR: R = −0.613, *p* < 0.001) [128]. The clinical impact of this discovery lies in highlighting the importance of the achievement of tight glycemic control which normalizes the antiremodeling effects of RAS-inhibition.

On the other hand, there is a clinical trial (NCT04401423, completed, phase 2) whose purpose is to identify the preventive effect of Ang-(1-7) against acute kidney injury, as well as the deterioration of multiorgan failure resulting from COVID-19 in severely affected patients. Participants will be exposed to a 3 h dose of TXA127 intravenously at 0.5 mg/kg per day for 10 consecutive days or until medical discharge. This trial demonstrated safe administration in critically ill COVID-19 patients. Additionally, it would be interesting and beneficial to document the effect of TXA127 in patients who have previously suffered acute myocardial infarction [129]. Interest in the effects of Ang-(1-7) is growing, so there are several ongoing clinical trials to evaluate its effects in different scenarios. To date, the results obtained in the different clinical trials show a promising future and a paradigm shift in the treatment of cardiovascular diseases, pointing to Ang-(1-7) and the MasR axis as new therapeutic targets for the reduction in the burden of cardiovascular morbidity and mortality.

## 6. Perspectives and Conclusions

It is important to investigate the new therapeutic strategies for the treatment of cardiovascular diseases, one of the main causes of death worldwide. Starting from the understanding of the overactivation of the classical RAS as a pathophysiological determinant in their progression is essential.

This review of the literature shows that the counter-regulatory RAS mainly ends with the synthesis of Ang-(1-7), Ang-(1-9), and alamandine. After binding to their specific receptors, these peptides activate multiple signaling pathways and cause beneficial effects on the cardiovascular system. Multiple studies have sought to analyze in vivo and in vitro the level of protection conferred by peptides of the counter-regulatory RAS; even though the evidence is convincing, there is still a long way to go. There are still several challenges such as improving techniques for its rapid detection in patient plasma and thus knowing the physiological levels and variations that can occur in a pathological context. These data are important for establishing doses for clinical trials. On the other hand, the short half-life of RAS counter-regulatory peptides has encouraged the development of a new field of research and innovation that consists of the development of more stable forms of these peptides or mimetic peptides. With respect to Ang-(1-9), a molecule was published in a patent application and consists in a peptidomimetic synthesized with D amino acids by inverting the original sequence of Ang-(1-9) (retro-enantio Ang-[1-9]) which retains the antihypertensive and cardioprotective activities of Ang-(1-9) [130]. By expanding knowledge about these peptides and innovating in forms of administration, we will move closer to novel therapies for use in clinical practice.

## Figures and Tables

**Table 1:** Main cardioprotective effects of the peptides of the counter-regulatory RAS.

Peptide	Cardioprotective Effects
Angiotensin- (1-9)	Prevention of cardiac hypertrophy in myocardial infarcted rats [102].
	Attenuation of cardiac fibrosis in stroke-prone spontaneously hypertensive rats [96].
	Cardioprotection against I/R injury via AT2R/Akt pathway in neonatal rat cardiomyocytes and isolated rat hearts [99].
	Prevention of cardiomyocyte hypertrophy by regulating mitochondrial dynamics in neonatal rat cardiomyocytes [97].
Angiotensin- (1-7)	Promotion of vasodilation via the activation of the MasR and NO–soluble guanylyl cyclase pathway in spontaneously hypertensive rats [81].
	Decreasing fibrosis in resistant arterioles via DUSP1/MAP kinase/Smad/CTGF signaling in rats with Ang II-induced hypertension [82].
	Reduction in cardiac fibrosis by inhibiting effect on KCa3.1 channels through ERK1/2 pathway, decreasing TGF-β1, and reducing collagen synthesis in mice treated with Ang II via osmotic mini-pumps [83].
Alamandine	Reduction in the accumulation of collagen and fibronectin in cardiac tissue of rats treated with isoproterenol [77,109].
	Reduction in the vascular expression of proinflammatory genes (CCL2, TNF-α, and IL-1β) in rats treated with doxorubicin [110].
	Prevention of Ang II-induced hypertrophy through AMPK/NO via the MrgD in neonatal rat cardiomyocytes [75].

## Data Availability

Not applicable.
